# Perspectives on shared decision making related to medications from patients with multiple long-term conditions transitioning from hospital to home: a qualitative study

**DOI:** 10.1007/s11096-026-02143-x

**Published:** 2026-04-21

**Authors:** Mikas Glatkauskas, Malin Olsen Syversen, Liv Mathiesen, Michael Scott, Karin Svensberg, Berit Gallefoss Denstad, Marianne Lea

**Affiliations:** 1 Research Group for Clinical Pharmacy, Department of Pharmacy, Section for Pharmaceutics and Social Pharmacy, University of Oslo, Oslo, Norway; 2 Medicines Optimization Innovation Centre, Antrim Area Hospital Site, Antrim, Northern Ireland UK; 3 Department of Pharmacy, Uppsala University, Uppsala, Sweden; 4 User Representative, Lillehammer, Norway; 5 Department of Clinical Pharmacy and Counselling, Oslo Hospital Pharmacy, Hospital Pharmacies Enterprise, South Eastern Norway, Oslo, Norway

**Keywords:** Decision making, Medication therapy management, Multimorbidity, Patient-centered care, Shared

## Abstract

**Introduction:**

Shared decision making is particularly important for patients with multiple long-term conditions due to the nature of long-term treatments and frequent changes in medication regimens. However, the complexity of the medication regimens could exclude these vulnerable patients from shared decision making. There is little knowledge about how patients with multiple long-term conditions experience and perceive shared decision making.

**Aim:**

The aim was to explore the perspectives and experiences of patients with multiple long-term conditions regarding shared decision making related to medications before, during and after a hospital stay.

**Method:**

Semi-structured interviews with 21 patients and three next of kin were conducted. Patients ≥ 18 years, usually living at home, on at least four medications for at least two separate conditions were included. These patients were purposively sampled from two geriatric wards and one internal medicine ward at a university hospital in Norway and interviewed approximately 14 days post hospital discharge. The inclusion and interviews lasted from December 2022 to February 2024. A semi-structured interview guide was used, and the qualitative data were analyzed using directed content analysis guided by the three-talk model developed by Elwyn et al. from 2017.

**Results:**

Patients reported not being invited to be part of the shared decision-making process and perceived their limited medical knowledge as a barrier to being invited to participate. They reflected on themselves being primarily focused on single details regarding one medication option they had received. Furthermore, they were not encouraged by the healthcare professionals to discuss and compare different medication options. Both patients and next of kin described an expectation that decisions being made by healthcare professionals would be accepted although the patient did not necessarily understand the treatment plan adequately. Several patients reported that healthcare professional led decisions left little to no room for further discussion and that medication decisions and patient health goals were almost solely in the hands of the healthcare professional. Although most patients trusted the healthcare professional to act in their best interests, this reliance resulted in further disengagement from their own treatment.

**Conclusion:**

Patients with multiple long-term conditions were in general unfamiliar with and uninvolved in shared decision making related to medications. Additionally, the patients reflected on a lack of invitation to team talk which resulted in limited patient involvement both in option and decision talk.

**Supplementary Information:**

The online version contains supplementary material available at 10.1007/s11096-026-02143-x.

## Impact statements

- There is a substantial gap between the experiences reported by patients with MLTCs and their next of kin regarding SDM and models of a patient-clinician partnership in decision-making.
- Healthcare providers should be encouraged to engage patients with MLTCs in discussions about all available treatment options rather than presenting one single option.
- Patients with MLTCs want to participate in discussions about how medication choices are made and how those choices will affect their lives after discharge; such discussions should occur before final decisions are made.

## Introduction

Shared decision making (SDM) is a collaborative, person centered process developed to arrive at preference and values based medical decisions surrounding a patient’s medications and other treatments [1, 2]. Research shows positive results when implementing the approach including outcomes such as increase in effectiveness, cost reduction and patient satisfaction [3, 4]. The concept of SDM began to appear in scientific literature and became a major theme in clinical communication research after Charles et al. publication in 1997 [5]. Although SDM is not yet common practice it has been introduced in different healthcare fields including geriatrics, oncology and pediatrics [4, 6, 7]. People with multiple long-term conditions (MLTCs) constitute a vulnerable and diverse patient group who often experience transitions of care, who manage complex medication regimens, and where decisions surrounding medications are frequent [2, 6]. Compared to treating one condition, patients with MLTCs must make decisions which encompass multiple goals surrounding the status of their different conditions [4, 6]. The principle of “no decision about me without me” and the use of SDM is especially relevant in persons with MLTCs, yet this ambition remains largely unrealized in practice [6, 8].

Involving the patient in decision making has been shown to lead to: increased patients’ medical knowledge, improved risk perceptions about different treatments, a greater number of decisions consistent with patient’s values, and fewer patients remaining passive or undecided [3, 4, 9]. The combination of MLTCs leads to more complex medication regimens, and this can often exclude patients with MLTC’s from SDM practice [6, 10]. During a period of illness, patients can be in a state of uncertainty, vulnerability and loss of power [4, 11]. SDM is advocated to be a tool that may assist a patient in restoring the autonomous capacity that they would otherwise have [6, 12, 13]. The three-talk model of Elwyn et al. [14], which revolves around “team talk”, “option talk” and “decision talk”, was developed in close collaboration with healthcare professionals (HCPs) to assist patients in navigating choice awareness, pros and cons with different options, and medical decisions based on “what matters most” to the patient [14].Based on a systematic review of multiple SDM models the three-talk model has inspired the creation of subsequent SDM frameworks, and the model has had significant impact on what constitutes the SDM-process [14, 15]. The concept of SDM shifts the power dynamics during the healthcare encounter between HCPs and patients to achieve a more balanced interaction [8]. SDM practice fits well to encompass the complexity of treating MLTCs, although how patients with MLTCs experience SDM practice during transitions of care is a less investigated phenomenon [6, 16].

### Aim

The aim of this study was to explore the perspectives and experiences of patients with MLTCs regarding SDM related to medications before, during and after a hospital stay.

## Method

### Study design

The research design was based on phenomenology using the SDM model developed by Elwyn et al. [14] as a framework for the interview analysis [14, 17, 18]. The data were collected during a larger research project exploring medication use during transitions of care [19]. This paper reports qualitative data in accordance with the standards for reporting qualitative research (SRQR) criteria [20].

### The authors’ background

The group of authors that contributed to the data collection and analysis had varying educational backgrounds and experiences. The group included both male and female authors with Northern, Western, and Eastern European backgrounds, a patient user representative and clinical pharmacists. The authors’ levels of experience in qualitative research ranged from limited to advanced. Throughout the group meetings, discussions were held to reflect on how the authors’ professional and sociocultural backgrounds, as well as their value systems, might have influenced the data analysis [21].

### Norwegian healthcare context

In Norway, patients receive all their medications from the hospital during their hospital stay; however, medications are not dispensed upon discharge. After leaving the hospital, the responsibility for managing medications shifts to the patient, and the general practitioner (GP) has the formal medical responsibility for the patient. The hospital physician prepares a discharge summary that should include a medication list, and which is delivered to both the patient and their GP. Typically, patients living at home visit their preferred community pharmacy to fill the prescribed medications after discharge [22, 23]. The municipality healthcare system includes homecare nursing (HCN) services, which can provide support with medication management, wound care, and personal hygiene [24]. Additionally, information about and participation in decisions around treatment is a fundamental right for patients in Norway [25].

### Participant inclusion

The inclusion of patients employed the purposeful sampling method to ensure a broad scope of sociodemographic factors including gender, age, education and ethnicity [26]. Patients were recruited from two geriatric wards and one internal medicine ward at a university hospital in Norway, with the interviews being conducted within 14 days after discharge. Patient inclusion took place from December 2022 to February 2024. Selection was based on predefined inclusion criteria, and guidance was provided by the HCPs on the wards in identifying potential patients. Patients were invited to participate in the study one to three days before their scheduled discharge, and informed consent was obtained. Approximately five days after discharge a study pharmacist contacted the patient via telephone to arrange the interview. Some patients were assisted by their next of kin with medication management tasks. Examples of such could have been filling prescriptions at the pharmacy, assistance with reading the discharge summary and ordering prescriptions from the GP. To enrich the data and improve our understanding of the challenges faced by the patients, next of kin were invited to participate if they assisted the patient with medication management post hospital discharge and if they were present at the patient’s home during the interview.

### Patient inclusion criteria

-  ≥ 18 years.
- Residential address in Oslo, Norway.
- Home-dwelling.
- Managing medications independently (may have some assistance from an HCN and/or next of kin).
- Using a minimum of four medications from a minimum of two therapeutic classes (ATC level 1).
- Minimum of two long-term conditions.

### Next of kin inclusion criteria

At home at the time of the interview and providing some assistance for the patient’s medication management after hospital discharge.

### Patient exclusion criteria (patients were ineligible if one or more criteria were met):

- Terminally ill.
- Isolated due to severe infections.
- Earlier participation in the study. (In case of readmissions).
- Advanced cognitive impairment (as assessed by the hospital physician).
- Not planned for discharge to their own home.
- Unable to communicate in Norwegian or English.

### Data collection

The interview guide was developed with input from the authors, hospital physicians and a medical scientist. The topics were developed based on the identified knowledge gap and the research groups’ earlier study [23] and focused on self-management, medication information, and SDM, where the latter was guided by the three-talk model [14, 27]. The interview guide (see Supplementary file 1) was constantly evaluated and revised twice (after the fourth and ninth interview) to deepen insights into specific themes during the data collection period. Interviewers (MG and MOS) received training in interviewing methodology by an experienced qualitative researcher (KS), e.g., how questions should be asked and elaborated.

Data were collected through semi-structured interviews [28]. Each participant was interviewed individually in their home, or at hospital, according to their preference. At the beginning of every interview a structured medication reconciliation was performed, and demographic variables were collected. Both the medication reconciliations and the interviews were audiotaped.

### Data analysis

The audio files were uploaded to NVivo v12. for transcription and qualitative coding. To achieve a broader understanding of the interview data, the conversation during the medication reconciliation was also included in the analysis. All but one interview was in Norwegian, and transcribed data were analyzed in Norwegian to ensure accuracy. The interview in English was coded along with the Norwegian data and incorporated into the final analytical text. The interview guide was broad and was used in relation to a larger research project exploring medication use during transitions of care [10, 29]. The three-talk model was used as the framework for the directed content analysis and helped search for specific topics surrounding SDM [14, 30, 31]. As described by Elwyn et al., the three elements of SDM are team talk, option talk and decision talk. Team talk is described as interactions where the patients and HCP identify goals, inform patients about the existence of a choice and the HCPs inviting the patient into the decision process. Option talk is mainly focused on the comparison of different treatment alternatives, the harms and benefits as well as informed discussions surrounding risks. Decision talk is explained by how patients and HCPs arrive at informed preferences so patients who receive treatment truly understand the choice, and that the choice is based on the wishes and values of the patient [14].

The researchers MG, ML and LM each reviewed two patient interviews individually according to the themes and discussed the categories during a consensus meeting. The initial three themes, (team talk, option talk and decision talk), were broken down into smaller categories that constituted the coding book for the analysis (see Supplementary file 2). The coding book was used as a guide to categorize the interviews after the three main themes in the three-talk model. Thereafter the main themes were broken down into more specific categories which in more detail helped mark the existence or absence of SDM. After the consensus meeting researcher MG individually categorized 10 additional patient interviews which were audited by ML and LM during two additional consensus meetings. As no substantial changes were made, author MG categorized the rest of the transcripts. Author MG selected representative quotations which were translated to English and discussed with authors ML, LM, and MS, before agreeing on which will be presented in the results and if the translations were correct. In the quotations presented the following abbreviations are used: I = Interviewer, P = Patient, PR = Patient relative.

Saturation was continuously evaluated during the data collection period reviewing the depth and richness of the findings as well as the diversity of the patients. To enrich the data, some patients were selected for follow up interviews after an additional 21–28 days. After 18 individual patient interviews, three patient and next of kin interviews and three follow-up patient interviews it was concluded that overall saturation had been reached [32, 33].

## Ethics approval

Written, informed consent from patients and next of kin was collected before inclusion in the study. De-identified data was immediately stored in a protected area for sensitive data (TSD) at the University of Oslo. The study was approved by the Regional Committee for Medical and Health Research Ethics (ref.no 420920/REK south-eastern C), Sikt (ref.no 919319), and by the data protection office at the university hospital. A small gift (value of 50 NOK – Four EUR or Five USD) was given to the patients after the interviews.

## Results

Interviews were conducted with 18 individual patients and three patients accompanied by their next of kin. Demographic data for patients and next of kin are presented in Table 1. The interviews were carried out at the patient’s home except for one that was undertaken in the hospital according to patient preference. The next of kin were all women, two of them were partners to the patient and one was a daughter of the patient. The interviews, including the medication reconciliation, took on average 95 min (± 22) and conducted on average 11 days (± 4.3) post hospital discharge. Additionally, to enrich the data, three follow-up individual patient interviews were conducted with an average length of 49 min (± 6), 34 days (± 2.5) after the first interview. The analysis revealed various insights on how patients experienced the three elements of SDM as described by Elwyn et al. 2017 [14]. The patients and the next of kin mainly reflected upon their most recent interactions with HCPs, predominantly related to their last hospital stay and post-discharge.

**Table 1:** Characteristics of patients and their next of kin in the analysis population

Characteristic—patients	21 patients
Age in years, median (range)	72 (22–94)
Female, number	11
Patients living alone, number	13
*Country of origin, number*	
Norway	18
Other (Scandinavian, European, African)	3
*Education, number*	
No university degree	12
University degree	9
No work experience in healthcare	18
Work experience in healthcare	3
Number of long-term conditions at discharge, median (range)	6 (2–13)
Number of medications in the discharge summary, median (range)	9 (5–21)
*Assistance with medication administration post-discharge, number*	
Manage themselves	13
Manage themselves with help from homecare nurse	5
Manage themselves with help from next of kin	2
Manage themselves with help from homecare nurse and next of kin	1
Characteristic – next of kin	3 next of kin
Female, number	2
Assist the patient with medication administration post-discharge, number	3

### Team talk

Most patients described a lack of invitation by the HCPs to be made aware of or to consider choices related to medications. Although some patients recalled being invited to participate, the invitation was perceived as non-authentic in terms of patients experiencing themselves as a less important partner rather than an equal collaborator.

“Being in hospital is similar to being in the military, you do as you are told to do” (Male age 86)

Furthermore, the patients recalled downplaying the importance of their own insights about their health. Instead, they perceived their own limited medical knowledge to be a barrier to meaningful collaboration. They experienced that this knowledge gap created distance between themselves and HCPs and hence prevented HCPs from extending invitations to patients to join conversations about medication decisions.

When asked about the need to be involved in decision making, the patients expressed a perception of being medically inexperienced. They did not describe HCPs who provided support towards the patient’s own health goals, in other words, the principle of being a team during this step of decision making was not highlighted during their communication with HCPs.

“…you must come back to the fact that you are in an unknown world. So, it becomes, even though you are invited to participate, you still become a sort of junior partner in the process.” (Male age 75).

For many of the patients, their perspectives on making a choice during hospitalization were also influenced by their health situation. Participation in a decision-making team was difficult for some patients who had limited energy due to their health condition during hospitalization. Furthermore, some patients reflected on the power imbalance between them and the HCPs in terms of gratitude for being in the hands of the HCPs, combined with not wanting to exert any more pressure by demanding some sort of involvement. One patient stated that she did not feel central to her own medication treatment and described feeling simply grateful to receive any form of assistance from the healthcare system.

“I don’t feel that I am a big part of my own treatment < … > you’re just happy to receive any type of help < … > you don’t get the feeling that you can demand any sort of meeting” (Female age 66).

Patients commonly described how their health goals and treatment plans were decided by the HCPs, rather than negotiated in a collaborative manner. Even when conversations took place, patients experienced that plans remained in the hands of the HCPs and that there was little room for any further involvement. The patient perspective of the vulnerable and stressful situation surrounding a hospital admission removes their ability to question the HCP decisions. In this context SDM-practice is not a priority for neither the patient nor the HCP.

### Option talk

The patient’s descriptions of how options were presented indicate that they were not invited to engage in comparing alternatives or communicating risks during their interactions with HCPs [14]. Instead, although making the patient feel informed, the patients described that the HCP simply listed the different treatments the patient was going to receive followed by a close-ended question if this sounded fine for the patient, rather than comparing treatment options based on the risks and benefits and providing evidence-based information. However, some patients perceived this situation as being part of the decision making.

“The physician was talking about an injection first that can take care of this heart flutter. < … > . If it would not work, we could try a weak electrical shock, she asked. < … > I think this sounded very good, and I was a part of the decision there. < … > I understood what it was about.” (Female age 66).

Furthermore, not every patient expressed a desire to or an understanding of to what extent they can be involved in the discussion of different options. For example, one patient articulated the importance of retaining control, yet was not interested in exploring medication alternatives, highlighting the differences in engagement levels between individual patients. The patients focused more on how they would accept the single option being presented to them by the HCP, rather than being invited to discuss different options. The patients described that their primary concern typically centered around the explanation of specific details regarding medications such as side effects, ways and time of administration and if the new medication had no interactions with the other medication on their list. Sometimes, this information was also not available for the patient, as expressed by one next of kin.

“Maybe they (the HCPs) could have given a better explanation of why you should be taking this and that medication and how much of each one” (Female age 81)

There were also patients who reflected on how discussing different treatment options and details can be overwhelming, citing potential information overload and risking that information is not remembered by the patient. This can indicate that HCP’s ways of explaining and comparing options are not being adapted to the patient’s knowledge level, again reflecting the medical knowledge gap between patients and HCPs.

“Yes, but you immediately can go into so many details (surrounding options). I believe that a lot of this type of information can be forgotten” (Male age 87).

The power imbalance was also apparent when it came to describing option talk. The patients expressed trust in the clinician expertise and described little awareness of anything other than to accept the medication recommendations proposed, indicating that the HCPs they communicate with had no perceived intentions of presenting different options and any details surrounding them:

“The physician recommends something, and I do not think that I have many other options than to say yes, but that is completely natural. He knows what they (the medications) are, and I believe that is fine”. (Male age 87).

### Decision talk

When asked about arriving at the final decision many patients reflected that they often reach a decision or express a preference without fully comprehending the information provided. Patients reported that they often agreed to HCP-led decisions without fully understanding of which consequences the decision will have for the patient’s daily life. An example was a patient who agreed to transition from a self-managed medication system using a pill organizer to a more automated multidose dispense system (MDD) without being able to foresee the real impact. This resulted in perceived loss of control of the names, shapes and colors of own medications. Here, the lack of decision talk led to loss of control and a stressful situation the patient must face on his own.

“Every Sunday, I could see and recognize my tablets inside and out. Now when they’ve taken over (the HCN with the MDD system), but when they started with the different milligrams, and the metoprolol just, if I’m not mistaken it changed the shape < … > I don’t know anymore which is which” (Male 76).

Some of the patients described having experienced genuine SDM. They gave examples of negotiation with the HCPs e.g. to avoid increasing the dose and/or to choosing alternative agents.

“We agreed together. I did not want to up the dose of candesartan, then we decided together that we will go forward and give amlodipine a try on the side.” (Female age 68)

Some decisions were perceived finalized prior to being communicated to the patient, that is leaving little room to raise concerns or suggest alternatives to the situation, although the solution was obviously impractical for the patient after discharge. This exemplifies how focusing on medication(s) rather than the patient can make patient values less important, and the principle of “what matters most to the patient” is lost.

“ I asked if it was necessary for me to take 10 tablets, will I not manage with less? < … > I got told that it had something to do with the pricing, or so he thought. So that was a bit stupid.” (Female 66).

On the other hand, conflicting with SDM, some patients were very thankful when situations regarding their health were handled “behind their backs”. This was viewed by some patients as a convenient way of handling the patient’s health status as well as something that reflected trust that the medical authorities are always working for the best ways to treat patients.

“My GP called me and informed me about a few poor metrics in my kidneys, and that he had called the hospital and that they were already waiting for me there, (laugh) < … > so that the GP feels responsible for you, that gives me trust.” (Male 77).

**Fig. 1:**
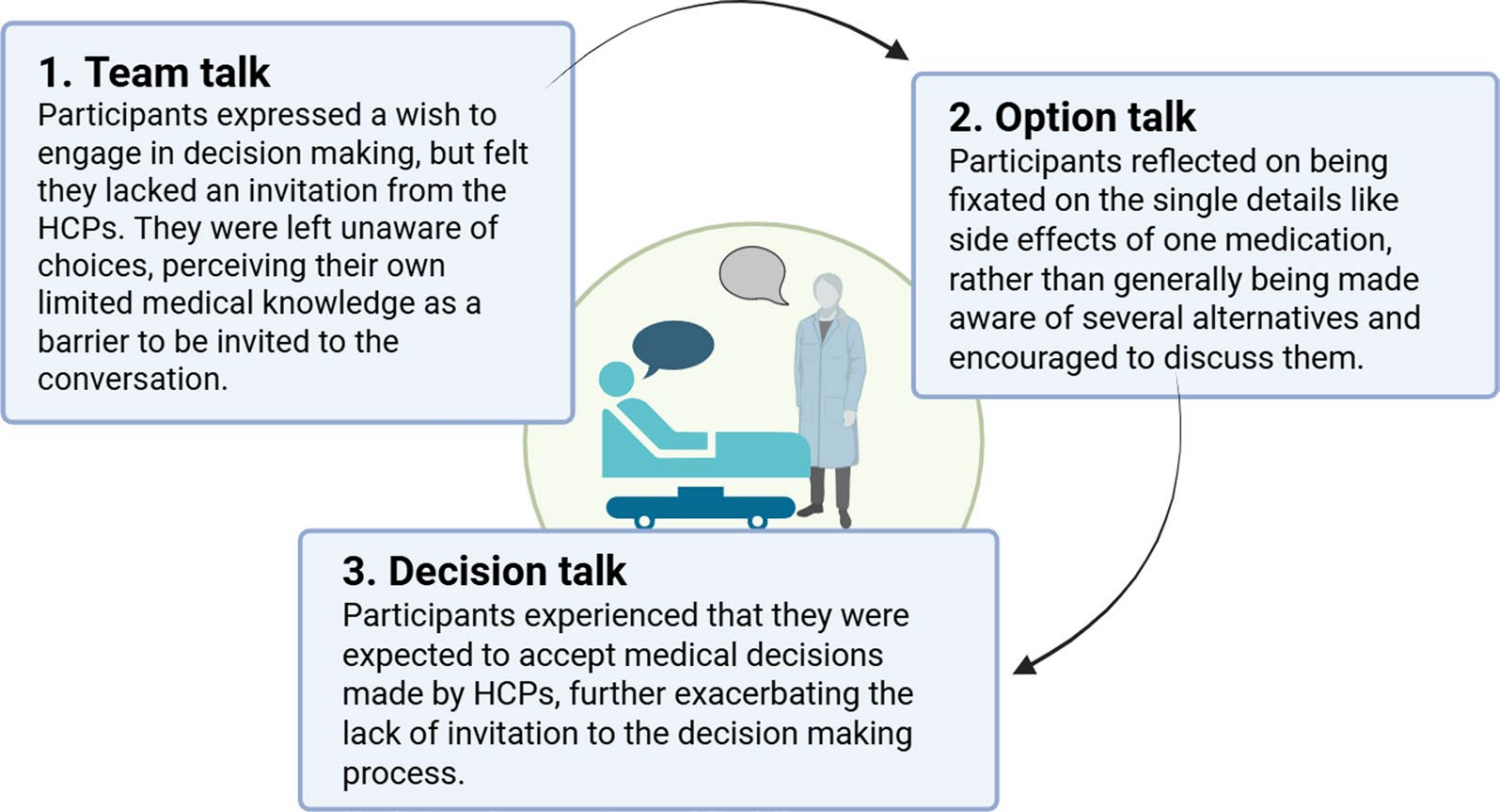
Summary of the key patient perspectives identified in patients with multiple long term conditions during the analysis. The summarized results are shown in the blue boxes. The curved arrows depict the timeline of the three talk model and how the consultation process shou ld progress. (HCPs Health Care Professionals

Figure 1 provides a view of the described results and summarized the key patient perspectives of each of the three SDM elements “team talk”, “option talk” and “decision talk”.

## Discussion

Utilizing the three-talk model we describe how the patients experienced limited knowledge about how to participate in SDM practice regarding medication decisions. The patients reflected on the lack of invitation to be a part of the decision by the HCPs ultimately resulting in passive acceptance of decisions. The patients identified their own limited medical knowledge to be a barrier for SDM, complementing the misconception of what SDM practice is about. Authority of and trust in the HCP often led to decisions being made with minimal patient involvement, which was sometimes viewed as convenient and trustworthy.

The patients reflected that they were rarely made aware of choices regarding medications when communicating with HCPs. Patients with MLTCs must manage complex medication regimens daily making this patient group particularly vulnerable when changes are being made to their medications [2, 34, 35]. The goal of team talk is to provide support to patients after they are made aware of choices and to use their goals to guide the decision making process [14]. Our results reflected that this support was not possible to achieve as the patients were not aware that they had the fundamental right to participate to begin with [25]. The patients reflected that their own limited medical knowledge was a barrier for participation, which is also reflected in earlier studies [36–39]. These results show how there is a lack of support for the patient and their next of kin, as the essence of SDM is to prove “the lack of medical school” to be irrelevant for the decision-making process [12, 36, 40].

The task of option talk should compare different alternatives, describing the potential benefits and harms of certain medications [14]. At best our patients reflected on the HCPs listing several treatments which the patient will undergo, rather than a comparison of alternatives. Our patients reflected on practical issues regarding medications upon returning home. We also revealed a passive acceptance of option decisions caused by a perceived reduced health status and autonomy. Although seemingly convenient, studies explain how HCPs premature recommendations deny the patient any form of deliberation in the process of choice awareness [9, 41]. Additionally, in vulnerable situations where the patient seemingly hands all decision making over to the HCP, it would help retain patient autonomy to invite a next of kin to participate [12, 42]. Fostering awareness of different options is linked to a better execution of other SDM steps, such as informing patients about different preferences, thus leading to improved medical outcomes and a better grasp of their complex medication regimens [36, 41, 43].

The patients reflected on decisions that were made by HCPs before taking the time to discuss what matters most to them. Previous research has documented time shortages as a barrier explaining why SDM might not be prioritized within treatment plans, despite its importance [2, 14, 44]. Our patients also expressed the thought of not wanting to appear intrusive, viewing themselves as junior partners in the decision-making process regarding their medications. Another study showed similar misconceptions about SDM as patients are unaware that they have a voice in the decision-making process [45]. This confusion leads to patients just being cooperative, simply showing gratitude as the HCPs are taking care of them, without considering if they are actually taking time to listen to “what matters most to you” [45]. If healthcare services aim to focus more on patient empowerment and SDM, a shift will need to happen.

### Strengths and limitations

Patients were interviewed in a comfortable environment, usually in the patient’s own home. This ensured that the interviewer was viewed as someone the patient could trust, and that their opinions would not have an influence on the medication treatment or the healthcare they were receiving. The interviews together with the medication reconciliation assured that the researchers spent adequate time learning about the patients, their health and home environment situation. In addition, we included next of kin where possible and conducted follow-up interviews to enrich the data. The prolonged interaction together with the research group’s extensive clinical experience provided a deep understanding to the interview data [46]. The interview guide directed the interviewers to topics focusing on medication management and shared decision making, but the conversations were also guided by the patients, maintaining reflexivity. Due to the purposive sampling the included 21 patients were of a wide demographic background and medical history [26]. Additionally, the patients were included from three different wards at a university hospital in Oslo, Norway. The diversity of the patient group, the analytical methods in addition to the length of the interviews ensured a rich amount of data which strengthens the transferability and trustworthiness of the results [47].

Although the interviews took place shortly after hospital discharge, our data were based on the patient’s memories of the encounters with HCPs which can introduce some memory bias into our results. The purposeful sampling of the patients assured a broad demographic background; however, the relatively long period of inclusion could have introduced different healthcare services to the patients. Despite the long time, services remained unchanged during the time of inclusion. We included a diverse sample of patients, however, it’s important to note that those who consented to participate may be more empowered and confident. This study primarily focused on patient reflections. Although we included three next of kin, more next of kin participants would have given a broader understanding of how patients experience SDM through the eye of the next of kin. We carried out three follow-up interviews which provided no further insight into the patient’s SDM experience post hospital discharge. It is possible that more follow-up interviews could have provided valuable information as well.

## Conclusion

Our findings revealed that in general patients were unfamiliar with SDM practice related to medications. Patients perceived lack of invitation from HCPs to engage in SDM, resulting in limited patient involvement in team talk, a limited discussion of options and decisions made without the patient necessarily understanding the treatment adequately.

## Supplementary Information

Below is the link to the electronic supplementary material.

Supplementary file1 (DOCX 55 KB)

Supplementary file2 (DOCX 22 KB)

## Data Availability

Due to the qualitative and sensitive nature of the dataset, full transcripts and audio files are not publicly available. De-identified materials (e.g. additional anonymized excerpts) are available from the corresponding author on reasonable request.
