# Supplementary material for: Perspectives on shared decision making related to medications from patients with multiple long-term conditions transitioning from hospital to home: a qualitative study
Source: Int J Clin Pharm. 2026 Apr 21;48(4):1636–45. doi: 10.1007/s11096-026-02143-x (PMC13368897; doi:10.1007/s11096-026-02143-x)
Supplement: Supplementary file 1 — Supplementary file1 (DOCX 55 KB) [file 11096_2026_2143_MOESM1_ESM.docx]

Self management:

- How do you feel that medication use works for you in your daily life?
  - How does it affect your life?
  - What practical challenges do you face?
  - What do you think works/does not work?
  - What could be done to make it easier/better?
- What does it mean for you to be responsible for medication management?
- What is important for you to know about the medications? Is there anything that is less important? What is important in daily life?
- What do you think is essential to be able to manage medications independently?
  - How do you involve yourself in your own medication use?
  - What do you think works/does not work?
  - To what extent did you feel that the hospital facilitated your ability to manage medications when you were discharged?
  - What can the hospital/healthcare system do differently to make you feel even safer?
- Have you seen your general practitioner after discharge? To what extent do you feel that the general practitioner facilitates your ability to manage medications? (Do they help you/availability)
- Who do you contact if you are uncertain?
- What do you think can increase quality of life regarding medication treatment? What do you think works/does not work?

Study nr.: ______ Start of the interviw (date/time): __________

Shared Decision-Making:

- Have you heard of shared decision-making?
  - What does shared decision-making mean to you?
  - Who should determine the goals for your treatment? How important is it for you to be involved in what type of medication you will use?
- Have you previously been presented with different options for your medication treatment?
  - If yes, do you have any examples?
  - How do you feel the communication has been tailored to you? Do you receive written information?
  - What information did you receive about the various options?
- How do you experience being involved in decisions regarding your medication treatment?
  - In the hospital? After discharge, for example, with your general practitioner?
  - What do you feel weakens your ability to take responsibility for your own treatment?

Medication Lists:

- How do you gather information about yourself and your medication treatment/use?
  - What do you think about the medication information in the discharge summary (epikrise)?
- What digital solutions do you use to obtain information about your medication treatment/use?
  - What are your thoughts on having access to digital solutions?
- Are you familiar with HelseNorge? What do you think about it?
- If there was a digital solution available to all your healthcare providers that included your medication list, what are your thoughts on that?

I would like to go through your medications.

Previously Published Interventions:

- What are your thoughts on follow-up conversations from the hospital after discharge? Would having an open option to call be helpful?
- What do you think about medication management support (LMG) after discharge?
- How do you feel about longer consultation times and digital follow-up?
- What do you think could have been done differently?
- Any other comments or suggestions?

We have now gone through the points I wanted to discuss with you today. Has it been alright for you? Is there anything you would like to add?

If there is anything that comes to mind afterward that you didn't get to say today, please feel free to contact me. Is it okay if I call you if there’s something I feel I didn’t get an answer to or didn’t ask about?

Thank you very much for taking the time to talk with me; it has been very helpful. Good luck moving forward!

- **Hoveddel**
- **Reconciliation**

Start with the introduction and follow the guide clockwise

**Semistructured patient and next of kin interview**

The purpose of this study is to gather knowledge and understanding of how to achieve medication treatment without unnecessary delays, based on the needs of the users. The results, along with findings from previous studies, will form the basis for a new, improved study that follows patients during their hospital stay and into primary healthcare.

The interview takes about one hour. If you wish to take a break or stop at any time, just let me know. If there are any questions you do not want to answer, that is completely fine. I would like to hear from you about how you experience and what you think about various topics related to medication treatment.

Ending

- **Introduction – aim and time**
