# Supplementary material for: Perspectives on shared decision making related to medications from patients with multiple long-term conditions transitioning from hospital to home: a qualitative study
Source: Int J Clin Pharm. 2026 Apr 21;48(4):1636–45. doi: 10.1007/s11096-026-02143-x (PMC13368897; doi:10.1007/s11096-026-02143-x)
Supplement: Supplementary file 2 — Supplementary file2 (DOCX 22 KB) [file 11096_2026_2143_MOESM2_ESM.docx]

**Supplementary file 2.**

Shared decision-making codebook: Based on Elwyn et al. 2017 three talk model [1].

SDM- recognizes the need to support autonomy by building good relationships, respecting both individual competence and interdependence on others.

**Main codebook used for qualitative coding.**

| **Theme** | Category | Description |
| --- | --- | --- |
| **Team talk**  **(Choice talk)** |  |  |
|  | Indicate choice | To inform the patient that a choice exists. To become aware of options. |
| Conveys awareness that a choice exists – initiated by either a patient or a clinician. This may occur before the clinical encounter | Provide support | “Let’s work as a team to make a decision” |
|  | Identify goals | Patient expressing their goals, concerns, fears (is this a known product?), and preferences. Active listening-is a big part. |
|  | Lack of team talk | The patient insists on giving the physician full responsibility. Additionally, the physician not involving the patient to join the team as the expert in their body. |

| **Theme** | Category | Description |
| --- | --- | --- |
| **Option talk** |  |  |
|  | Discuss harms and benefits | Explanation of harms and benefits  Side effects and so on |
| Patients are informed about treatment options in more detail | Compare alternatives | What are the different alternatives of treatment |
|  | Risk communication | Associated risks with the treatment |

| **Theme** | Category | Description |
| --- | --- | --- |
| **Decision talk** |  |  |
|  | Get to informed preferences | The knowledge provided is considered good enough to understand the differences |
| Patients are supported to explore “**what matters most to them**”, having become informed. | Make preference-based decisions | “Tell me what matters most to you for this decision” |
|  | Get to not so informed preferences | Initially the patient believed they received enough information, but it turned out to be not so when actually using the medication |
|  | Patients decide more than the physician | The patient lacks support from the physician when making a decision. |
|  | Physician making decisions without the patients notice | The patient describing a decision which was made prior to informing the patient |

References:

1. Elwyn G, Durand MA, Song J, et al. A three-talk model for shared decision making: multistage consultation process. BMJ. 2017;359.<https://doi.org/10.1136/bmj.j4891>.
